# Stepwise organ-preserving management of incidental pT1 rectal adenocarcinoma: outcomes after completion transanal endoscopic microsurgery (TEM)

**DOI:** 10.1007/s00464-025-12313-z

**Published:** 2025-10-20

**Authors:** Alberto Arezzo, Carlo Alberto Ammirati, Giovanni Distefano, Michele Barbiero, Simone Arolfo, Roberto Passera, Mario Morino

**Affiliations:** 1 Department of Surgical Sciences, University of TurinCittà Della Salute E Della Scienza Hospital, Corso Bramante 88, 10126 Turin, Italy; 2 Division of Nuclear Medicine, University of Turin Città Della Salute E Della Scienza Hospital, Turin, Italy

**Keywords:** pT1 rectal cancer, Transanal endoscopic microsurgery (TEM), Endoscopic resection, Organ preservation, Salvage surgery

## Abstract

**Background:**

Accurate in vivo assessment of rectal lesion invasion remains challenging despite advances in high-definition endoscopy and AI-assisted diagnostics. Some lesions resected endoscopically for presumed superficial pathology are ultimately found to contain submucosal invasive adenocarcinoma (pT1), prompting reconsideration of treatment. While total mesorectal excision (TME) remains the standard for radical oncologic removal, its morbidity has increased interest in organ-preserving approaches such as transanal endoscopic microsurgery (TEM).

**Methods:**

We conducted a retrospective, single-centre study including all consecutive patients who underwent TEM after endoscopic mucosal resection (EMR) or endoscopic submucosal dissection (ESD) of rectal lesions unexpectedly diagnosed as pT1 adenocarcinoma. Patients treated between 1995 and 2024 with at least 12 months of follow-up were included. Primary endpoints were overall survival (OS) and disease-free survival (DFS); secondary endpoints included residual disease in the TEM specimen and patterns of recurrence.

**Results:**

Sixty-six patients were included. TEM identified residual dysplasia in 25 patients (37.9%) but no cases of residual invasive carcinoma. Surgical margins were clear in all cases. Only one patient (1.5%) required salvage TME due to adverse histological features. At a median follow-up of 15 months, OS was 100% and DFS 97%, with two patients (3%) experiencing local recurrence successfully managed with salvage surgery. No distant metastases were observed. No stoma formation or major complications occurred.

**Conclusions:**

In patients with incidental pT1 rectal adenocarcinoma following EMR or ESD, completion TEM provides excellent short-term oncological outcomes with minimal morbidity. This two-step, organ-preserving approach appears oncologically adequate in well-selected low-risk patients and offers a viable alternative to radical surgery, especially when maintaining function is a priority.

Despite significant advances in endoscopic imaging and in vivo histological assessment, the risk of underestimating the actual depth of neoplastic invasion during standard endoscopic procedures remains considerable, as even in expert hands, lesions that appear to be confined to the mucosa are sometimes found to contain submucosal invasion upon final pathology [1–3]. This discrepancy between endoscopic impression and histological reality presents a crucial challenge in managing early rectal neoplasia, especially when patients are initially considered for endoscopic resection only.

Endoscopists often face diagnostic uncertainty when assessing superficially appearing lesions, particularly flat or sessile rectal adenomas. As a result, some patients initially managed endoscopically are unexpectedly diagnosed with invasive pT1 adenocarcinoma upon final pathology, requiring further intervention [4].

From an oncological perspective, the incidence of nodal metastases in pT1 rectal adenocarcinoma ranges from 6 to 20%, influenced by additional histopathological factors, including poor differentiation, lymphovascular invasion, tumour budding, and submucosal invasion depth [5–7]. Consequently, the traditional recommendation has been radical resection via total mesorectal excision (TME), which enables en bloc removal of the tumour and mesorectal lymphadenectomy, aiding both staging and treatment. Nonetheless, TME is associated with substantial morbidity, including risks of urinary and sexual dysfunction, defaecatory disorders, and permanent stoma, particularly in elderly or comorbid patients [8].

These considerations have driven increasing interest in less invasive, organ-preserving strategies. With the advent of endoscopic mucosal resection (EMR) and endoscopic submucosal dissection (ESD), early rectal neoplasms can often be removed en bloc, allowing for precise histopathological evaluation [9]. However, when submucosal invasion is unexpectedly detected, clinicians must choose between completing radical surgery and performing secondary, local transanal excision. The latter, often employing transanal endoscopic microsurgery (TEM), aims for complete full-thickness excision at the initial resection site, potentially removing residual neoplastic tissue and improving margin status while sparing patients the functional costs of radical surgery [10, 11].

The rationale for this “stepwise local management” is both oncological and patient-centred: it enables histologically complete excision when endoscopic margins are positive or uncertain and provides a high-quality assessment of residual disease, and it offers a less morbid, tailored approach. In clinical practice, this method might strike the best balance between oncological safety and quality of life, especially in patients with low-risk histological features.

However, the oncological adequacy of local re-excision alone remains a matter of debate. While retrospective studies and small prospective series report promising outcomes in carefully selected low-risk tumours [12, 13], concerns remain about the risk of undertreatment, particularly when nodal metastases are undetected. The omission of lymphadenectomy limits the accuracy of staging, which can impact subsequent decisions regarding adjuvant therapy and the intensity of surveillance.

Furthermore, the biological behaviour of pT1 adenocarcinomas is heterogeneous. Some tumours may possess aggressive molecular profiles or microscopic lymphatic involvement that goes unnoticed without lymph node sampling. Therefore, careful patient selection, based on both endoscopic features and histopathologic risk factors, remains essential.

Ultimately, the key clinical question is: can transanal re-excision after endoscopic removal of early rectal cancer deliver oncological outcomes comparable to radical surgery, particularly regarding local recurrence, disease-free survival, and overall survival? Furthermore, what criteria best identify patients who might benefit from this conservative approach without compromising long-term oncological results? This study aims to address these important issues in a real-world cohort of patients with pT1 rectal adenocarcinoma identified after EMR or ESD and subsequently treated with TEM.

## Material and methods

### Study design and population

This is a retrospective observational study including all consecutive patients who underwent transanal endoscopic microsurgery (TEM) as a completion excision following prior endoscopic resection (EMR or ESD) of rectal lesions that were found to harbour pT1 adenocarcinoma at final histopathology. Patients were treated between 1995 and June 2024 at the Department of Surgery of the University of Turin, a tertiary referral centre for colorectal surgery and advanced endoscopy. In this series, all patients with pT1 rectal cancer after EMR/ESD were treated with TEM, and no patient underwent primary TME in this setting.

Inclusion criteria were as follows:

- Initial resection of a rectal lesion using EMR or ESD.
- Histological diagnosis of pT1 adenocarcinoma in the endoscopic specimen.
- Subsequent local excision using TEM was carried out within 12 weeks of the initial endoscopic resection.
- Availability of comprehensive follow-up data for at least 12 months.

Patients with synchronous colorectal cancer, who underwent primary surgical resection without prior endoscopic therapy, or who presented with metastatic disease at diagnosis, were excluded.

### Data collection

Demographic data (age, gender), endoscopic characteristics of the index lesion (location, size, and distance from the anal verge proximally and distally), histological findings from both endoscopic and TEM specimens, and details regarding follow-up and recurrence were collected retrospectively from institutional databases. Tumour location was classified as lower (0–5 cm), middle (6–10 cm), or upper rectum (11–15 cm from the anal verge). Lesion size was determined by the largest diameter reported endoscopically. Pathological review of all specimens was performed by specialised gastrointestinal pathologists in accordance with international guidelines.

In case of persistent neoplasia, histopathological parameters collected included margin status, depth of invasion, tumour grade, the presence of lymphovascular invasion, and tumour budding. The resection from TEM was considered curative if there was no residual carcinoma and negative resection margins.

### Outcome measures

The primary outcomes were overall survival (OS) and disease-free survival (DFS), defined as the time from TEM to death from any cause and to the occurrence of local or distant recurrence, respectively. Secondary outcomes included the rate of residual disease in the TEM specimen, the need for salvage surgery, and recurrence patterns (local vs. distant).

Recurrence was assessed through scheduled clinical and radiological follow-up, including digital rectal examination, flexible sigmoidoscopy, carcinoembryonic antigen (CEA) levels, pelvic MRI, and total body CT scan at 3, 6, and 12 months, and yearly thereafter, in line with national surveillance guidelines.

### Statistical analysis

Descriptive statistics were used to summarize patient demographics, tumour characteristics, and outcomes. Continuous variables were expressed as medians and interquartile ranges (IQRs). Categorical variables were reported as absolute and relative frequencies. Kaplan–Meier survival curves were constructed for OS and DFS. Statistical analysis was performed using R version 4.5.1 (R Foundation for Statistical Computing, Vienna, Austria).

The study was conducted in accordance with the Declaration of Helsinki and approved by the institutional review board (IRB) of Azienda Ospedale Università—Città della Salute e della Scienza di Torino. Given the retrospective design, the need for written informed consent was waived.

## Results

### Patient and lesion characteristics

**Fig. 1:**
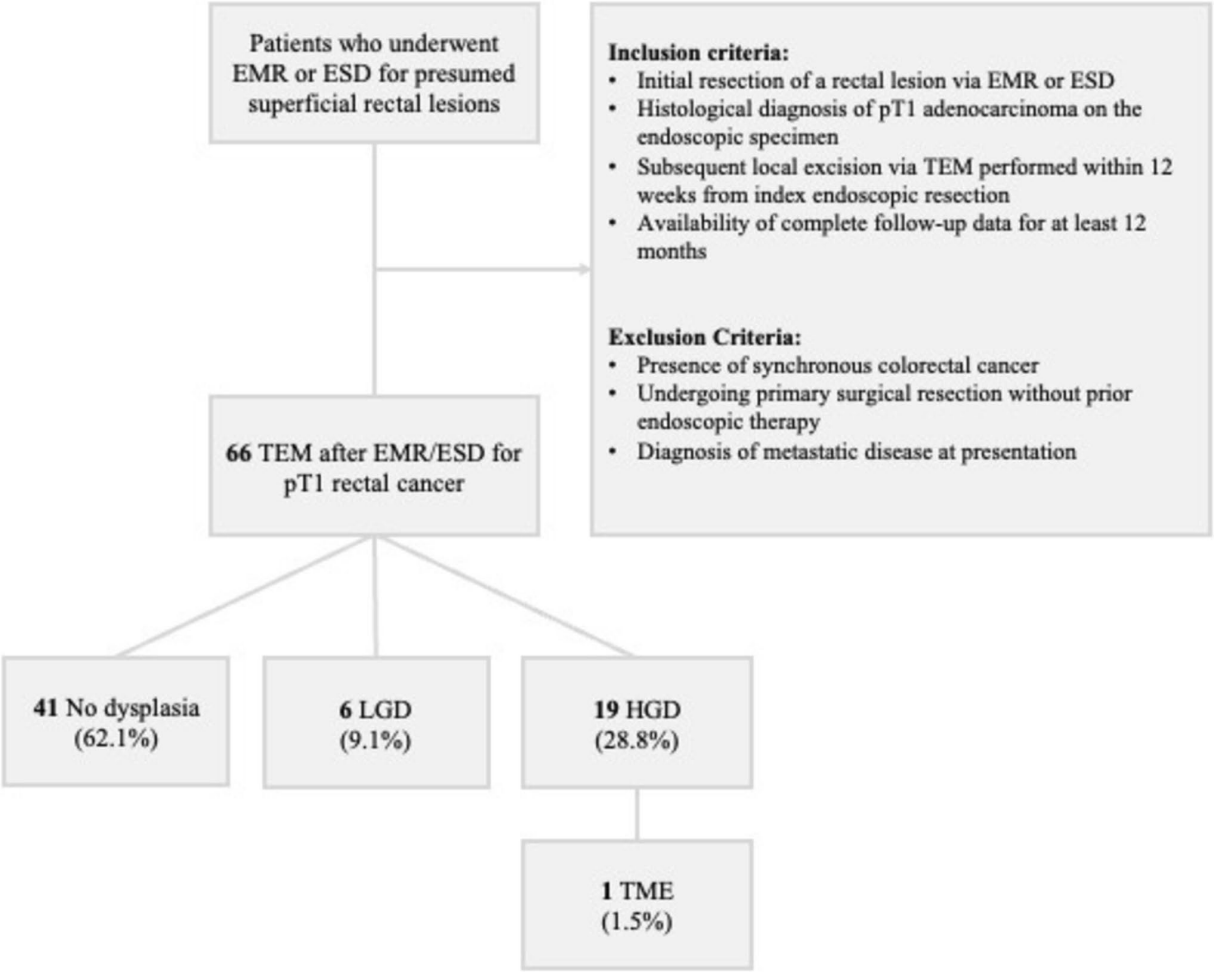
Flow diagram of patient inclusion and study design. *EMR* endoscopic mucosal resection, *ESD* endoscopic submucosal dissection, *LGD* low-grade dysplasia, *HGD* high-grade dysplasia, *TEM* transanal endoscopic microsurgery, *TME* total mesorectal excision

A total of 66 patients were included in the analysis (Fig. 1). Patients’ characteristics are displayed in Table 1. The median age was 68 years (IQR 59–74) with a slight predominance of male patients (59.1%, n = 39). Most lesions were located in the middle rectum (63.6%) or lower rectum (19.7%), with only a minority involving the upper rectum or rectosigmoid junction. The median tumour size at the time of endoscopic resection was 2 cm (IQR: 2.0–3.0 cm). The median distal margin from the anal verge was 7.7 cm (IQR: 5.0–10.0), and the proximal margin was 10.3 cm (IQR: 8.0–12.0).

**Table 1:** Characteristics of patients

Variable	Value
Median age, y [IQR]	68 (59–74)
Gender (M/F)	39 (59.1%) / 27 (40.9%)
Tumour location (Upper/Mid/Lower)	11 (16.7%) / 42 (63.6%) / 13 (19.7%)
Lesion size, cm [IQR]	2 [2, 3]
Distal margin, cm [IQR]	7.7 [6–8, 10–12]
Proximal margin, cm [IQR]	10.3 [10–14]

*M/F* male/female, *IQR* interquartile range, *cm* centimetres, *y* years

All patients underwent either endoscopic mucosal resection (EMR) or endoscopic submucosal dissection (ESD) for an initially suspected superficial lesion. Histological analysis of the endoscopic specimen showed invasive adenocarcinoma confined to the submucosa (pT1) in all cases. None of the patients had synchronous malignancies or distant metastases at the time of diagnosis.

### Histopathological findings after TEM

Following the initial EMR/ESD, all patients underwent a second local excision using full-thickness transanal endoscopic microsurgery (TEM). The indication for TEM was histological evidence of submucosal invasion in the original endoscopic specimen, often with uncertain or involved margins, or patients with high-risk features and comorbidity that did not meet the criteria for radical surgery.

Histopathological analysis of the TEM specimen revealed residual neoplastic tissue in 25 cases (37.9%). Among these, 19 lesions showed low-grade dysplasia (LGD), and 6 lesions showed high-grade dysplasia (HGD). No cases of residual invasive adenocarcinoma were found. Surgical margins were clear in all cases. None of the patients had lymph node staging, as no radical proctectomy was performed after TEM, except for one patient who underwent salvage surgery due to adverse histological features detected at the TEM specimen (high-grade and lymph vascular invasion).

### Surgical and oncologic outcomes

Surgical and oncologic outcomes are displayed in Table 2.

**Table 2:** Follow-up of patients

Variable	Value
Histology after TEM	
No dysplasia	41 (62.1%)
LGD	6 (9.1%)
HGD	19 (28.8%)
Residual invasive cancer	0
Salvage surgery performed	1 (1.5%)
Local recurrence	2 (3%)
Distant recurrence	0
Median follow-up, [IQR] mos	15 [12–48]
Median OS, years	9.7
12-month OS	100%
12-month DFS	97%

*TEM* transanal endoscopic microsurgery, *LGD* low-grade dysplasia, *HGD* high-grade dysplasia, *OS* overall survival, *DFS* disease-free survival, *mos* months

The procedure was completed in all patients without requiring conversion to open surgery or necessitating intraoperative assistance. No major intraoperative complications were reported.

One patient (1.5%) underwent salvage surgery, a low anterior resection with TME, due to the presence of HGD and tumour budding in the TEM specimen. Final pathology after proctectomy showed no residual tumour and no lymph node involvement.

At a median follow-up of 15 months (IQR: 12–48 months), only two patients (3%) experienced local recurrence. No distant metastases were identified in the study population. Both local recurrences occurred at the site of the original lesion and were detected during routine follow-up endoscopy, confirmed by imaging. These patients were referred for salvage surgery.

Kaplan–Meier analysis showed that overall survival (OS) was 100% at 12 months, with no deaths from any cause during the follow-up period. The median OS was 9.7 years; ten patients died during the entire follow-up. Disease-free survival (DFS) was 97% at 12 months. The two patients who experienced recurrence remained alive and disease-free after salvage intervention, highlighting that the recurrence rate observed after TEM in this setting is comparable to that reported after radical surgery, while TEM ensures equivalent oncological outcomes with the additional benefit of preserving anorectal function.

### Oncologic efficacy

Overall, the two-step approach, combining EMR/ESD followed by TEM, enabled complete oncological control in the vast majority of patients. The absence of residual invasive carcinoma in the TEM specimens indicates that the initial endoscopic resection was already oncologically adequate in a substantial proportion of patients. The addition of a local full-thickness re-excision likely contributed to the low recurrence rates observed.

The proportion of patients with residual dysplasia after endoscopic resection—almost 38%—underscores the importance of TEM in detecting and removing microscopic disease that may have been left behind. Notably, this approach spared most patients the morbidity associated with radical resection, while delivering excellent functional outcomes and preserving the integrity of the anorectal anatomy.

### Functional and morbidity considerations

Although functional outcomes were not systematically evaluated in this study, no patient required temporary or permanent stoma formation, and no cases of major postoperative complications (Clavien-Dindo ≥ III) were documented. Anecdotally, most patients regained their baseline bowel habits within weeks of the procedure, emphasising the minimally invasive and organ-preserving character of TEM.

### Surveillance and recurrence patterns

All patients underwent standard postoperative surveillance, including endoscopic and radiological follow-up. The early detection of the two local recurrences within 12 months enabled prompt salvage intervention, emphasising the importance of close monitoring in patients treated with local excision strategies.

No recurrence was observed beyond 12 months, although longer follow-up is required to confirm the durability of these results. No patient in this cohort developed distant recurrence, supporting the hypothesis that a subset of pT1 rectal cancers may have limited metastatic potential if carefully selected.

## Discussion

This study supports the feasibility, safety, and potential oncological adequacy of transanal endoscopic microsurgery (TEM) as a completion treatment following endoscopic resection of rectal lesions unexpectedly diagnosed as pT1 adenocarcinoma. In our cohort of 66 patients, a two-step, organ-preserving strategy combining endoscopic mucosal resection (EMR) or endoscopic submucosal dissection (ESD) followed by full-thickness local excision via TEM resulted in excellent short-term oncological outcomes. The 12-month overall survival (OS) and disease-free survival (DFS) rates were 100% and 97%, respectively. Only two cases of local recurrence (3%) were observed. These results align with emerging evidence supporting tailored, function-preserving approaches in managing early rectal cancer [12–14].

Accurate histological staging remains essential in determining the optimal treatment for rectal neoplasms. Despite advances in endoscopic imaging, such as narrow-band imaging, chromoendoscopy, magnification, and computer-aided diagnosis, differentiating between mucosal and submucosal invasion in vivo remains challenging [1–3]. Consequently, lesions removed endoscopically for suspected high-grade dysplasia or intramucosal carcinoma are sometimes found to be invasive upon final pathology. For pT1 adenocarcinoma—particularly when high-risk histologic features are present—radical proctectomy with total mesorectal excision (TME) remains the gold standard [5, 6, 15]. However, TME is associated with significant morbidity, including risks of bowel, urinary, and sexual dysfunction, especially in elderly or comorbid patients [16].

Within this clinical setting, full-thickness local excision via TEM provides an attractive alternative for patients with pT1 rectal cancer who are either unsuitable for radical surgery or do not exhibit high-risk features. In our series, 38% of patients had residual dysplasia in the TEM specimen (19 LGD and 6 HGD), yet none showed residual invasive carcinoma. These findings emphasise the dual diagnostic and therapeutic roles of TEM, particularly in evaluating residual neoplasia and histological markers of aggressiveness, such as lymphovascular invasion and tumour budding [10, 12, 13]. These factors are often complex to assess accurately in fragmented EMR specimens.

The low recurrence rate and absence of distant metastases in our study suggest that omitting lymphadenectomy in carefully selected patients (no high-grade or lymphovascular invasion at TEM specimen) may be a justified approach. Although the risk of lymph node metastasis in pT1 rectal cancer can range from 6% to over 20% depending on histological risk factors [7, 17], this risk is significantly lower in patients with low-risk features. Indeed, several retrospective studies and meta-analyses have shown that local excision—whether as a primary treatment or as a step towards radicalisation after EMR/ESD—can achieve oncological outcomes comparable to those of TME in such patients [10, 18–20]. Considering the effective oncologic outcome, a non-operative management with strict follow-up can be argued. This remains a debated issue within the scientific community, and our choice to manage all pT1 lesions operatively with TEM reflects the current lack of high-level evidence supporting a non-operative strategy in this setting.

Notably, the two patients who experienced local recurrence in our study were identified during routine follow-up and successfully treated with salvage surgery. This highlights the importance of strict surveillance protocols in patients managed with local excision alone, particularly when lymph node assessment has not been performed. Structured follow-up allows for the early detection of recurrences and increases the likelihood of successful salvage treatment, thereby emphasising the safety of organ-preserving approaches when combined with vigilant monitoring.

In terms of functional outcomes, TEM clearly provides advantages over radical surgery. In our cohort, no patient required a temporary or permanent stoma, and no significant perioperative complications were reported. Although we did not formally evaluate quality-of-life metrics, existing literature shows that patients undergoing TEM generally experience faster recovery and better preservation of bowel function than those undergoing TME [21–23]. For elderly patients or those with limited physiological reserves, avoiding the morbidity linked to TME while ensuring oncologic safety is a meaningful clinical goal.

However, our study has limitations. The retrospective, single-centre design may introduce selection bias and limit the wider applicability of our findings. While our cohort was consistent in terms of treatment approach and follow-up, we did not stratify patients based on all individual histological risk factors, which could have improved the accuracy of predictive models. Additionally, long-term follow-up beyond 36 months is lacking, and late recurrences cannot be ruled out. The limited follow-up should be considered given the lack of available clinical information, particularly for patients treated in the initial years of implementation of this technique. Finally, quality-of-life and functional outcomes were not prospectively measured—an important area for future research.

Despite these limitations, our results contribute to the expanding evidence supporting minimally invasive, personalised strategies for early rectal cancer. With advances in endoscopic technology and growing expertise in histologic risk stratification, a more customised approach to the extent of surgical intervention is becoming increasingly feasible. Patients with low-risk pT1 lesions and clear margins after EMR/ESD may safely undergo completion TEM instead of TME, especially when preserving function is important. Conversely, radical surgery remains suitable when TEM uncovers residual invasive disease or high-risk features.

Future research should include multicentre randomised controlled trials comparing long-term oncological and functional outcomes of completion TEM versus upfront TME in patients with incidental pT1 cancer following endoscopic resection. Furthermore, integrating molecular, genomic, and immune biomarkers could enhance risk stratification and guide treatment decisions. Multidisciplinary collaboration among endoscopists, colorectal surgeons, pathologists, and oncologists is essential to ensure optimal, patient-centred care. Furthermore, improvements in intraoperative staging of rectal cancer at the time of local excision, such as the use of fluorescence to better characterise tumour histology and invasiveness [24], or sampling of potential sentinel lymph nodes in the mesorectum [25, 26], could significantly alter the perspective on organ-sparing techniques.

In conclusion, our real-world experience demonstrates that TEM, used as a secondary procedure following EMR or ESD for pT1 rectal adenocarcinoma, is a safe and effective option for carefully selected patients. It offers favourable short-term oncological outcomes and preserves rectal function without the need for radical resection. This approach is a valuable addition to the evolving landscape of personalised rectal cancer management and should be regarded as a viable alternative in organ-sparing treatment strategies.
